# The reliability of a frailty index may depend on the deficits included

**DOI:** 10.1007/s41999-022-00734-1

**Published:** 2023-01-11

**Authors:** Anna Martine Petronella Verwiel, Martin Pulvermann, Nadine Heleen Smit, René Johannes Franciscus Melis

**Affiliations:** Department of Geriatric Medicine, Radboud University Medical Center, Geert Grooteplein Zuid 10, 6525 GA Nijmegen, The Netherlands

**Keywords:** Frailty index, Older persons, Reliability, Validity

## Abstract

**Purpose:**

Recently, a short version of the TOPICS-MDS questionnaire, the Short Form (SF) was developed. The aim of this study was to assess the validity and reliability of two versions of a frailty index (FI) based on this SF: with or without information on present diseases included.

**Methods:**

This was a clinical, prospective study of geriatric patients. An FI based on the TOPICS-SF items alone (SF-FI) and an FI with the disease information as additional deficits (MDS-FI) were calculated. Criterion and construct validity and reliability were assessed.

**Results:**

95 patients were included. A strong correlation was found between SF-FI and MDS-FI scores (Pearson’s *r* = 0.92). Data supported four out of six hypotheses for the construct validity. However, a Bland–Altman plot and a regression analysis showed that the SF-FI was generally higher than the MDS-FI.

**Conclusion:**

This study indicates that the reliability of the TOPICS-SF-FI depends on the presence of disease information.

## Key summary points

**Aim:**

To assess the validity and reliability of a frailty index (FI) with (MDS-FI) or without (SF-FI) chronic diseases as deficits included.

**Findings:**

Despite a strong correlation between SF-FI and MDS-FI scores, further analysis showed the SF-FI was systematically higher than the MDS-FI.

**Message:**

When measuring an FI it is important to carefully choose deficits. As some items may be more correlated than others, this may over- or underestimate the true frailty.

## Introduction

Identification of frailty among elderly has relevance for clinical decision-making and contributes to personalized care. Several frailty indices (FI) have been derived from aging databases. In previous research, a standard procedure for creating an FI was developed showing that frailty variables can be selected at random; the specific nature of the variables is not important, as long as they cover a range of systems [1, 2]. Recently, a short version of a questionnaire based on the ‘The Older Persons and Informal Caregivers Survey Minimum Data Set’ (TOPICS-MDS) [3, 4], the TOPICS-Short Form (TOPICS-SF) was developed and validated as a patient-reported outcome measure (PROM) in clinical settings [5]. We established if and how an FI based on this short form can be calculated. While medical health is covered in the PROM itself through an item on subjective health, information on the presence of chronic diseases is not. An accompanying case mix form is used to collect disease information from the medical record. The feasibility of the FI would increase if the score could be established from the self-reported information alone. The aim of this study was to assess the validity and reliability of this SF-FI with and without disease information.

## Methods

This was a clinical, prospective study including geriatric in- and outpatients of 65 years and older at a university hospital in the Netherlands. We recruited a consecutive sample of patients and obtained the recommended sample size for this type of studies [6]. Exclusion criteria were: (1) not being able to answer on questionnaires due to severe cognitive impairments or a decreased level of consciousness, (2) requiring end-of-life care with a life expectancy of less than 2 weeks.

Complete TOPICS-MDS data were collected in an interview for the self-reported information, and by abstracting the present diseases and other case mix data from electronic medical records by a researcher. We then calculated an FI based on the 17 self-reported TOPICS-SF items alone (SF-FI). As a reference standard, we used the TOPICS FI based on the complete TOPICS-MDS (MDS-FI), which includes disease information on 18 chronic conditions and 7 extra items on aspects of well-being and health, amounting to a total of 42 items [4]. Data on relevant clinical and demographic variables (age, sex, and living situation) were collected, because they are previously found to be predictors of FI scores [4, 7]. The multi-morbidity was additionally operationalized as a Charlson co-morbidity index as established from the electronic health record by a researcher. The Clinical Frailty Scale (CFS) was registered by the geriatrician during comprehensive geriatric assessment on a 9-point scale (higher is more frail) [8].

Descriptive statistics were performed. To test criterion validity, a Pearson correlation analysis between the SF-FI and the MDS-FI was performed. To test the construct validity, SF-FI scores were examined according to predefined hypotheses (Table 1) for its correlation with patient characteristics such as age, sex, living situation and multimorbidity, using Spearman correlation analyses and independent sample *t* tests. We considered the SF-FI as having acceptable criterion and construct validity respectively, when the Pearson’s *r* for the correlation between the SF-FI and the MDS-FI was greater than 0.7 and at least 75% of our results were in correspondence with our hypotheses. This is in accordance with the COSMIN quality criteria for measurement properties [6, 9, 10]. To investigate the reliability of the SF-FI, the agreement between SF-FI scores and MDS-FI scores was visualized with a Bland Altman analysis. All statistical analyses were carried out using SPSS Statistics 25.

**Table 1:** Hypotheses to assess construct validity of the TOPICS-SF-FI

Hypotheses construct validity	
1	The SF-FI scores are significantly higher in women compared to men
2	The SF-FI scores are weakly to moderately correlated (*r* > 0.3) with increasing age
3	The SF-FI scores are significantly higher in patients admitted at the geriatric ward than in outpatient clinic patients
4	The SF-FI scores are significantly higher in patients who live in an institutionalized facility than patients who live at home independently
5	The SF-FI scores are weakly to moderately (*r* > 0.3) correlated with Charlson co-morbidity index scores
6	The SF-FI scores are moderately (*r* > 0.5) correlated with Clinical Frailty Scale scores

## Results

**Fig. 1:**
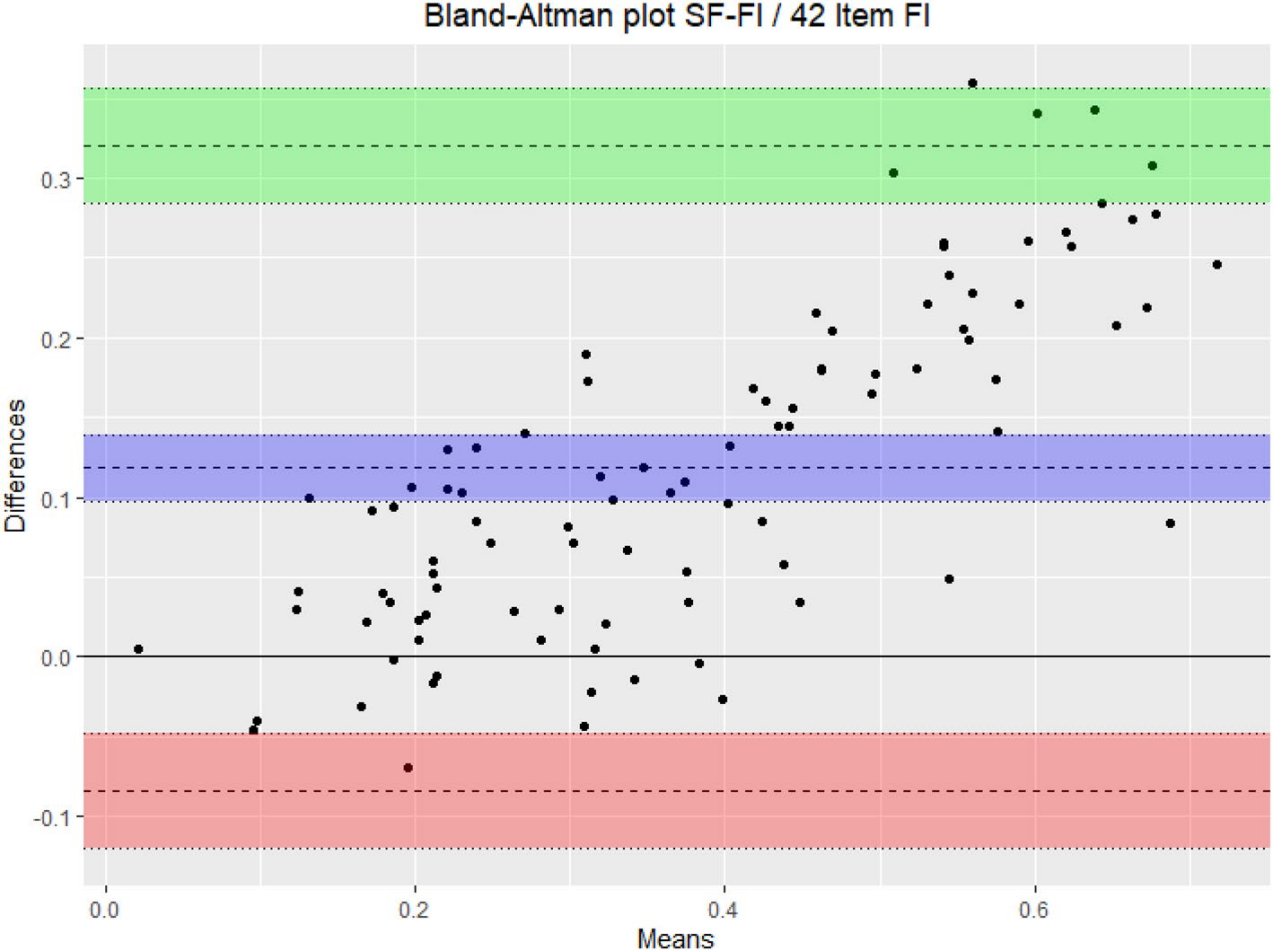
Bland–Altman plot: levels of agreement between SF-FI and MDS-FI. Lines with blue shading: mean difference with 95% confidence interval. Lines with red shading: lower limit of agreement with 95% confidence interval. Lines with green shading: upper limit of agreement with 95% confidence interval

A total of 95 elderly patients were included in the study. The mean (± standard deviation) age was 81 (± 7) years, a slight majority (55%) were women. A strong correlation was found between SF-FI and MDS-FI scores (Pearson’s *r* = 0.92). Data supported four out of six hypotheses for the construct validity, which means that two third of our results complied with the a priori defined hypotheses. Investigating the reliability, the mean SF-FI score was remarkably higher than the mean MDS-FI score [0.44 (0.21), respectively 0.32 (0.14)]. The mean difference between the SF-FI and the MDS-FI was 0.12 (95% confidence interval 0.10 to 0.14). A Bland–Altman analysis showed that the interval of the levels of agreement between the SF-FI and the MDS-FI was − 0.08 to 0.32 (Fig. 1). Regression analysis showed that the difference between the two frailty indices increased significantly with increasing mean of the MDS-FI and SF-FI (*R*^2^ of 0.61, *B* = 0.47, *p* < 0.001).

## Discussion

We showed that the construct validity between the SF-FI without morbidities and the MDS-FI is strong, but the reliability of the SF-FI was not satisfactory. Especially at higher frailty levels, the SF-FI scores overrated the frailty compared to the MDS-FI. This suggests that the excluded morbidity items make a considerable contribution to establish a reliable FI score and cannot be excluded. A possible explanation for the higher SF-FI scores lies in the nature of the excluded variables. The SF-FI contains mainly self-reported variables, whereas the MDS-FI also contains more objective disease information. Of the self-reported SF-items, 10 are (I)ADLs, and different (I)ADLs are more likely to co-occur than different morbidities. Additionally, it is likely that an FI based on a limited number of items (17 items) is more sensitive to a misbalance between the type of items included. While PROMs need to be short to be feasible and the ability to measure frailty based on self-reported PROM data continues to make sense, it is important to recognize that disease information (the casemix form accompanying the TOPICS-SF) cannot be skipped when calculating an FI. Further curtailment of a frailty index is not recommendable.

## Conclusions

This study indicates that the reliability of the TOPICS-SF-FI is not sufficient when excluding information on morbidities. When measuring an FI it is important to carefully choose deficits as some items may be more correlated [(I)ADLs] than others (morbidities). This may over- or underestimate the true frailty, while conceptually, the type of deficits accumulated may not matter.

## Data Availability

Available upon request.

## Code availability

Available upon request.
